# Maximizers’ Reactance to Algorithm-Recommended Options: The Moderating Role of Autotelic vs. Instrumental Choices

**DOI:** 10.3390/bs13110938

**Published:** 2023-11-16

**Authors:** Kaeun Kim

**Affiliations:** Department of Business Administration, Dong-A University, Busan 49236, Republic of Korea; kaeunkim@dau.ac.kr

**Keywords:** maximizing, satisficing, algorithmic decision-making, autotelic choices, instrumental choices

## Abstract

The previous literature has provided mixed findings regarding whether consumers appreciate or are opposed to algorithms. The primary goal of this paper is to address these inconsistencies by identifying the maximizing tendency as a critical moderating variable. In Study 1, it was found that maximizers, individuals who strive for the best possible outcomes, exhibit greater reactance toward algorithm-recommended choices than satisficers, those who are satisfied with a good-enough option. This increased reactance also resulted in decreased algorithm adoption intention. Study 2 replicated and extended the findings from Study 1 by identifying the moderating role of choice goals. Maximizers are more likely to experience reactance to algorithm-recommended options when the act of choosing itself is intrinsically motivating and meaningful (i.e., autotelic choices) compared to when the decision is merely a means to an end (i.e., instrumental choices). The results of this research contribute to a nuanced understanding of how consumers with different decision-making styles navigate the landscape of choice in the digital age. Furthermore, it offers practical insights for firms that utilize algorithmic recommendations in their businesses.

## 1. Introduction

In today’s rapidly evolving digital landscape, AI-assisted algorithm recommendations have become ubiquitous across various domains, profoundly impacting consumers’ choices. From personalized movie suggestions on streaming platforms to tailored product recommendations on e-commerce websites, algorithm-recommended options have revolutionized how consumers navigate many choices. This trend becomes particularly noticeable in the age of information abundance, where algorithms harness vast datasets to curate options that align with individual preferences. Examples of algorithm-recommended options can be found in platforms such as Netflix, which suggests movies based on viewing history, and Amazon, which tailors product recommendations based on browsing behavior and purchase history.

Backing this assertion are numerous studies and reports that highlight the increasing prevalence of algorithm-recommended options in our daily lives. According to Twilio [1], AI-driven personalization is utilized by over 90% of businesses to boost business growth. Also, the market size of recommendation engines is projected to expand from USD 5.17 billion in 2023 to USD 21.57 billion by 2028 [2].

However, despite the growing acceptance and integration of algorithm-recommended options, consumer attitudes toward these suggestions are not universally positive. Previous research has presented a complex and sometimes contradictory picture. On one hand, there is evidence suggesting that consumers appreciate and value algorithm-recommended options. For instance, Logg et al. [3] found that laypeople relied more on advice from algorithms than humans when making numeric estimates. This “algorithm appreciation” phenomenon was observed across various domains ranging from weight estimation to more subjective domains, such as forecasting song popularity and romantic attraction [3]. Also, findings from Banker and Khetani [4] suggest that individuals often rely too much on algorithms, even when they recommend inferior options, in a way that could threaten their welfare.

Conversely, consumers often exhibit aversion and reactance toward algorithm-recommended options. In fact, the majority of previous research has documented the “algorithm aversion” effect in which consumers generally prefer humans to algorithms [5,6,7,8,9,10,11,12,13]. For example, Promberger and Baron [5] showed that patients exhibited higher trust in a human physician compared to a computer program when accepting medical recommendations. Individuals perceived thorough discussions among humans as more valuable and fairer than mathematical formulas when selecting employees [7]. Also, while recommender systems perform objectively better than humans in predicting people’s preferences for jokes, consumers are reluctant to depend on such systems, partly because they find algorithm processes difficult to understand [13].

Intriguingly, these contradictory findings highlight the need to delve deeper into the underlying factors influencing consumer reactions to algorithm-recommended options. Previous studies have explored several boundary conditions that could explain under which circumstances consumers are more or less appreciative and aversive of algorithm-recommended options. Task objectivity plays a crucial role in which consumers trust algorithms more than humans for more quantifiable and measurable tasks, such as predicting stock prices and analyzing data [14]. Interestingly, when romantic partner recommendation, a seemingly subjective task, was depicted as an objective task, trust in algorithms (vs. a professional matchmaker) was higher compared to when it was described as a subjective task [14]. Additionally, acceptance of algorithm-recommended options was higher for utilitarian products than hedonic products [15]. Experts did not rely on algorithmic guidance as much as laypeople did [3]. Cultural dimensions also play a role in moderating algorithm aversion. The aspects of uniqueness neglect in algorithms intensified algorithm aversion among individualistic cultures, such as South America, while familiarity with algorithms diminished algorithm aversion among collectivistic cultures, like India [16]. Furthermore, consumers who are politically more conservative-leaning tend to evaluate AI-based services poorly [17].

Building on previous research, the goal of this paper is to examine a new factor—consumers’ distinct decision-making styles (maximizing and satisficing)—and how they can influence consumers’ responses to algorithm-recommended options. Findings from the two quantitative studies suggest that maximizing consumers exhibit greater reactance to algorithm-recommended options compared to satisficing consumers. Moreover, this reactance among maximizing consumers is observed when the choice goal is autotelic rather than instrumental. The present paper contributes to a nuanced understanding of how consumers with different decision-making styles respond to algorithmic recommendations and provides practical implications for firms that utilize algorithmic recommendations in their businesses. 

The rest of the paper is structured as follows. First, I provide a theoretical foundation that explores the connection between consumers’ maximizing tendency and reactance to algorithms. In the subsequent sections, I present two empirical studies that collectively support the proposed hypotheses. Finally, the general discussion section summarizes the findings, addresses theoretical contributions and managerial implications, and discusses the limitations and directions for future research.

### Maximizing and Reactance to Algorithms

Maximizing refers to a behavioral tendency of striving for the most optimal choice, whereas satisficing (i.e., satisfy and suffice) refers to a behavioral tendency of choosing and being satisfied with a “good enough” option [18,19]. A typical maximizer invests additional time and explores a broader array of options in order to arrive at the best decision [20,21,22], generally leading to objectively better decision outcomes compared to satisficers [23].

Do maximizers and satisficers respond differently to algorithm-recommended options? For maximizers who aim to optimize their decisions by extensively evaluating alternatives, the appeal of algorithmic suggestions lies in the potential for attaining the best possible outcome. If an algorithm can provide a recommendation that aligns with their pursuit of excellence, maximizers might be inclined to accept these suggestions.

Contrary to what one might expect, I argue that maximizers would, surprisingly, show greater resistance to choices suggested by algorithms when compared to satisficers. This argument builds upon prior research, which indicates that the act of making choices carries intrinsic meaning for maximizers [18,24,25]. In essence, the very process of choosing holds significance for maximizers. As Schwartz et al. [18] argued, maximizers view their choices as a reflection of their own identities. Since choice is tantamount to self-identity for maximizers, receiving negative feedback about their choices can potentially damage their self-concept and, consequently, lead to increased cognitive dissonance [26].

Moreover, in contrast to satisficers, maximizers are more likely to experience higher levels of eudaimonic happiness in which pursuing meaning and purpose is essential [24]. As such, the pursuit of the best choice serves as a device for finding existential meaning for maximizers [25]. Kokkoris [25] found that maximizers construe choice as an identity construction process, and thus, when such free choice is limited under situations like COVID-19, they feel more restricted and irritated.

Given their desire to actively shape their choices as a means of self-expression and identity construction, maximizers might resist algorithmic recommendations that appear to undermine their agency in decision-making processes. Thus, it is expected that reactance to algorithm-recommended options will be greater for maximizers than for satisficers. To formally state this hypothesis:

**H1.**  *Maximizers will show greater reactance to algorithm-recommended options than satisficers.*

Furthermore, such reactance to algorithm-recommended options will have downstream consequences on behavioral intention. If maximizers show greater reactance to algorithm-recommended options, they are less likely to adopt algorithms and are more inclined to make choices independently. Therefore, it is expected that algorithm adoption intention will be lower for maximizers than for satisficers due to their greater reactance to algorithm-recommended options. To formally state this hypothesis:

**H2.**  *Maximizers will have lower algorithm adoption intention than satisficers via increased reactance to algorithm-recommended options.*

It is also expected that the degree of maximizers’ reactance to algorithm-recommendation options would depend on the nature of the choice context—autotelic versus instrumental. A choice is autotelic when it “derives its meaning and purpose from within, from the act of choosing itself ([27], p.74).” For example, when a consumer chooses a movie just to enjoy, it is an autotelic choice. In comparison, a choice is instrumental when it “derives its meaning and purpose from the outside the act of choosing ([27], p.74).” Choosing a movie to write a critique for a literature class project is an example of an instrumental choice.

Previous research has identified that maximizers (vs. satisficers) not only value autotelic goals but also experience greater satisfaction from autotelic choices than instrumental choices [27]. When a choice is autotelic, where the intrinsic motivation of choosing itself matters, maximizers will construe a choice process as a means of reflecting their self-identity and finding existential meaning. Because letting algorithms choose on behalf of themselves can hamper maximizers’ self-concept, they are more likely to exhibit reactance to algorithm-recommended options. In contrast, when a choice merely serves as a pathway to attaining different goals, maximizers will not construe such instrumental choice as an identity construction process and, thus, will care less about algorithms making a choice instead of themselves. Therefore, it is anticipated that maximizers’ reactance to algorithm-recommended options will be higher when the choice goal is autotelic rather than instrumental. To formally state this hypothesis:

**H3.**  *Maximizers’ reactance to algorithm-recommended options will be higher in the autotelic choice condition compared to the instrumental choice condition.*

Two empirical studies were conducted to test H1–H3.

## 2. Study 1

The objective of Study 1 was to provide initial evidence that maximizers show greater reactance to algorithm-recommended options compared to satisficers (H1) and that this reactance would subsequently affect adoption intention of algorithm-recommended options (H2). To this end, Study 1 employed a quantitative survey method to examine the relationships between maximizing tendency, reactance to algorithm-recommended options, and algorithm adoption intention using pre-existing scales.

### 2.1. Participants

One hundred and fifty-one participants (*M*_age_ = 37.32; 53% male) were recruited via Prolific, and they completed an online study in exchange for USD 1.40. The amount of monetary compensation was determined in accordance with Prolific’s hourly wage guidelines, which is USD 12 per hour.

### 2.2. Procedures

Participants were instructed that the purpose of the study was to explore consumers’ decision-making styles and that they would be asked to answer a series of questionnaires. First, the degree of maximizing was measured with a 7-item maximizing tendency scale (e.g., “No matter what it takes, I always try to choose the best thing”; α = 0.86; [28] see Appendix A). Next, participants indicated their attitudes toward algorithm-generated options. For those who were not familiar with what an algorithm is, the following instruction was given to participants: “In the context of decision-making or recommendation systems, algorithm-generated options are those proposed by an algorithm, typically based on analysis of user data, patterns, or other factors, with the aim of providing relevant or tailored choices to individuals. For example, when you shop online, the AI algorithm recommends products you might like based on your search history.” Reactance to algorithm-recommended options was measured with a 5-item reactance scale adapted from Kokkoris [25] (e.g., “Because of algorithm-recommended options, I often feel very restricted as a consumer”; α = 0.95; see Appendix A). Both the maximizing tendency scale and reactance scale were answered on a 7-point scale (1 = strongly disagree, 7 = strongly agree). Finally, participants indicated their preference for algorithm-generated vs. personal choices on a 7-point scale (1 = choice made entirely by the AI algorithm, 7 = choice made entirely by myself) along with demographic measures.

### 2.3. Results and Discussions

Descriptive statistics and correlations with each of the focal constructs are presented in Table 1.

Supporting H1, maximizing tendency was positively correlated with reactance to algorithm-recommended options, *r* = 0.26, *p* < 0.01.

To test whether maximizers’ reactance has a downstream consequence on behavioral intention (H2), a mediation analysis using the PROCESS macro was conducted (model 4 with 5000 bootstrapped resamples [29]). First, maximizing tendency significantly predicted reactance, *b* = 0.37, SE = 0.11, *p* < 0.01. Next, when controlling for the maximizing factor, reactance significantly predicted choice preference, *b* = 0.36, SE = 0.07, *p* < 0.001. While the direct effect of maximizing on choice preference was not significant (b = −0.14, SE = 0.10, 95% CI= [−0.34, 0.06]), the mediation analysis revealed a significant indirect effect of maximizing on choice preference through reactance, with an effect size of 0.13 (SE = 0.05, 95% CI = [0.06, 0.25]), supporting H2.

The results from Study 1 show that those high in maximizing tendency exhibit greater reactance to algorithm-recommended options, which ultimately increases the preference for self-made choices rather than algorithm-made choices.

## 3. Study 2

The objective of Study 2 was to further illustrate that maximizers’ reactance to algorithm-recommendation options is heightened when the nature of choice is autotelic rather than instrumental (H3). To achieve this, Study 2 manipulated the choice goal by instructing participants to imagine choosing a product they either really liked (autotelic goal) or wanted to learn about (instrumental goal). Therefore, Study 2 was designed as a two-cell quantitative experiment.

### 3.1. Method

One hundred participants (*M*_age_ = 36.09; 47% male) were recruited from Prolific, and they completed an online study in exchange for USD 1.70.

### 3.2. Procedures

Participants were instructed that the study consisted of two parts: a decision-making style questionnaire and a product choice task. In the first part of the study, participants completed a Maximizing Tendency Scale (α = 0.85) as in Study 1 on a 7-point scale (1 = strongly disagree, 7 = strongly agree).

In the second part of the study, participants were given a choice scenario in which they had to choose a cheese. Depending on the choice condition, participants were randomly assigned to either the autotelic choice or instrumental choice. Following the procedures from the previous studies [27,30], a choice goal was manipulated by varying the instructions as to why participants chose a particular cheese. Specifically, participants in the autotelic condition were asked to imagine that they were browsing an online cheese website to find a cheese they *really liked* and were reminded that it was important that they could select an option they *really wanted* when making decisions. Participants in the instrumental condition were instructed to envision themselves exploring an online cheese website to *gather information* about various cheeses. They were also reminded of the importance of *being well-informed about the available options* when making decisions. After reading the instructions, ten different types of cheeses, along with their names, pictures, and information, such as milk type, texture, and flavor, were presented in a randomly vertical display order (see Appendix B for an example).

Participants were then informed that the website utilized AI technology to offer personalized cheese recommendations. They were asked to rate their likelihood of using the AI algorithm for obtaining cheese recommendations on a 7-point scale (1 = not at all likely, 7 = very likely). Reactance to the algorithm was assessed with two items: “Algorithm-recommended options are disturbing” and “Algorithm-recommended options are intrusive (α = 0.88)” on a 7-point scale (1 = strongly disagree, 7 = strongly agree) adapted from Bleier and Eisenbeiss [31] and Edwards et al. [32]. Finally, participants indicated their choice of cheese (choice conditions did not affect participants’ choice of cheese, *χ*(9)^2^ = 12.76, *p* = 0.17) and completed demographic questions.

### 3.3. Results and Discussions

Descriptive statistics and correlations with each of the focal constructs are presented in Table 2.

**Figure 1:**
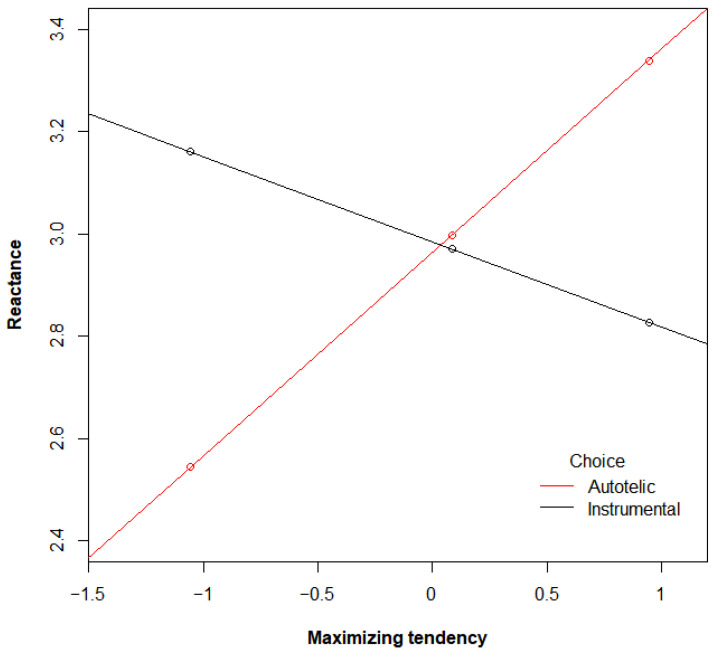
Interaction effect between maximizing tendency and choice goal on reactance.

To test H3, a moderation analysis using the PROCESS macro was conducted (model 1 with 5000 bootstrapped resamples, [29]). The maximizing tendency, choice condition, and interaction term (mean-centered) were regressed on reactance to algorithm-recommended options. The main effects of the maximizing tendency (*b* = 0.13, SE = 0.14, *p* = 0.37) and choice condition (*b* = −0.02, SE = 0.29, *p* = 0.95) were not significant. Supporting H3, a significant interaction effect between the maximizing tendency and choice condition emerged, *b* = 0.56, SE = 0.28, *p* < 0.05 (Figure 1). Specifically, in the autotelic choice condition, reactance to algorithm-recommended options increased as the maximizing tendency increased (*b* = 0.40, SE = 0.17, 95% CI = [0.06, 0.74]). In the instrumental choice condition, however, the maximizing tendency did not predict reactance (*b* = −0.17, SE = 0.22, 95% CI = [−0.61, 0.27]).

To further test whether such maximizers’ reactance to algorithm-recommended options for autotelic choices influences algorithm adoption intention (H2), a mediation analysis in the autotelic choice condition using the PROCESS macro was conducted, as in Study 1 [29]. First, maximizing tendency significantly predicted reactance, *b* = 0.40, SE = 0.15, *p* < 0.01. Next, when controlling for the maximizing factor, reactance significantly reduced algorithm adoption intention, *b* = −0.61, SE = 0.16, *p* < 0.001. The mediation analysis revealed a significant indirect effect of maximizing on algorithm adoption intention through reactance, with an effect size of −0.24 (95% CI= [−0.58, −0.05]). The direct effect of the maximizing on algorithm adoption was not significant (*b* = 0.36, SE = 0.18, 95% CI = [−0.01, 0.73]).

Replicating the results from Study 1, Study 2 shows that maximizers indeed exhibited higher reactance to algorithm-recommended options (supporting H1), which negatively affects the intention to adopt the algorithm (supporting H2). However, this effect was observed only when the choice goal was autotelic (i.e., the choice is the end goal) but not when it was instrumental (i.e., the choice is merely a means to achieve different goals), supporting H3.

## 4. General Discussion

The present research shows that maximizers, characterized by their pursuit of the best possible choice and extensive evaluation of alternatives, emerged as a critical factor in the algorithmic decision-making landscape. Contrary to the expectation that maximizers, given their pursuit of excellence, would be more inclined to accept algorithm-recommended options, findings from the current study suggest that maximizers paradoxically exhibit greater reactance toward algorithm-recommended choices than satisficers. This is because choice represents not only an optimization process but also a means of identity construction for maximizers. When algorithms step in to make decisions on their behalf, maximizers perceive this as a limitation on their autonomy and an obstruction to their search for meaning through choice. Furthermore, the present study highlights the importance of the choice context, distinguishing between autotelic and instrumental choices. In the case of autotelic choices, where the act of choosing itself is intrinsically motivating and meaningful, maximizers are more likely to experience reactance to algorithm-recommended options. In contrast, such reactance to algorithms was not observed among instrumental choices where the decision is merely a means to an end.

### 4.1. Theoretical Contributions and Practical Implications

From a theoretical standpoint, the current research contributes to the nuanced understanding of how consumers respond to algorithmic recommendations. The initial paradox highlighted in the previous literature—the coexistence of algorithm appreciation [3,4] and algorithm aversion [10,14,16]—has raised the need to examine boundary conditions of when and why consumers are more or less averse to algorithmic decision-making. Our results identified differences in decision-making approaches (maximizers vs. satisficers) and the nature of the choice goal (autotelic vs. instrumental) as two important boundary conditions. In addition, since the initial scholarly discussion about maximizing and satisficing by Schwartz et al. [18] dates back more than 20 years ago, little is known about how maximizers and satisficers behave in the modern digital era. Our findings shed light on the effect of algorithmic decision-making on maximizers and satisficers.

Our findings also provide practical and managerial implications for businesses and service providers that utilize algorithmic recommendations. First, recognizing the presence of maximizers within their user base, companies should be cautious when implementing algorithms, especially for choices that customers view as meaningful in themselves. Businesses can develop algorithms to detect users’ decision-making styles and provide tailored algorithmic recommendations. For example, rather than directly offering algorithm-suggested choices to maximizers, it would be better to highlight how algorithms can help them make the best choice and express self-identity.

Second and relatedly, businesses can better understand the influence of the choice context in which algorithm-recommended choices are utilized. In autotelic contexts, where consumers seek intrinsic satisfaction from choosing, companies should prioritize providing options that align with individual preferences and emphasize the role of algorithms less. In instrumental contexts, where consumers focus on achieving specific goals, companies could propagate the role of algorithms.

Finally, considering the general tendency of algorithm aversion among consumers [5,6,7,8,9,10,11,12,13], businesses can invest in user education initiatives to demystify recommendation algorithms. Providing users with insights into how algorithms work and how they benefit from personalized suggestions can mitigate reactance and foster trust in algorithmic recommendations.

### 4.2. Limitations and Future Research Directions

Although this paper provides valuable insights into the relationship between decision-making styles, choice contexts, and their interactive effects on reactions to algorithm-recommended options, it is important to acknowledge several limitations that may inspire future research directions. First, from a methodological perspective, the current study assessed behavioral intention using the hypothetical choice scenario. Because Studies 1 and 2 did not directly measure the actual adoption of algorithm-recommended options, future research could incorporate measures of actual adoption to bridge the gap between intention and behavior. For instance, the Weight on Advice (WOA) measures, as utilized in previous studies [3,33], could be integrated to assess whether maximizers indeed place less reliance on algorithmic advice in practical decision-making scenarios.

Second, maximizing comprises two facets: the pursuit of the best possible choice (goal) and an extensive search for alternatives (search) [34]. Depending on which of the two factors dominates, maximizing individuals could experience positive or negative affective states [35,36,37,38]. For example, Kim [38] found that the search aspect of maximizing is related to seeking both negative and positive customer reviews after product choice, which decreases the level of satisfaction. However, the goal aspect of maximizing is related to seeking positive customer reviews after product choice, and this does not decrease consumers’ satisfaction. While the current research did not differentiate between these facets of maximizing in relation to reactance toward algorithms, future research could delve deeper into the nuanced aspects of maximizing to determine which facet, or potentially both facets, drives maximizers’ reactance to algorithmic recommendations.

Last but not least, future research could delve into the differences in satisfaction between choices made by the maximizing individuals themselves and choices recommended by algorithms. Previous research suggested that maximizers experience greater satisfaction with autotelic choices than instrumental choices [27]. It is likely that maximizers may also find greater satisfaction in their own choices as opposed to algorithm-chosen options, particularly in autotelic choice contexts. Therefore, future research could employ experimental designs to explore whether maximizers indeed express higher satisfaction with self-made choices than algorithm-recommended choices in autotelic contexts while showing no significant satisfaction gap in instrumental choice contexts.

## 5. Conclusions

The goal of this paper was to address the mixed findings in the previous literature regarding whether consumers appreciate or are averse to algorithms by identifying maximizing tendency as a critical moderating variable. The results from the two quantitative studies supported the hypotheses that (1) maximizers exhibit greater reactance to algorithm-recommended options than satisficers, (2) this increased reactance has downstream consequences on algorithm adoption intention, and (3) maximizers’ reactance to algorithm-recommended options is higher when the choice goal is autotelic rather than instrumental.

In conclusion, by identifying maximizers as a critical segment of consumers who exhibit reactance to algorithm-recommended options, this research contributes to a nuanced understanding of how consumers with different decision-making styles navigate the landscape of choice in the digital age and offers practical insights for firms that utilize algorithmic recommendations in their businesses.

## Figures and Tables

**Table 1:** Descriptive statistics and correlations (Study 1).

Construct	M	SD	r		
Maximizing	4.53	1.04	1		
Reactance	3.48	1.48	0.26 **	1	
Choice preference	5.26	1.34	−0.01	0.37 **	1

** *p* < 0.01.

**Table 2:** Descriptive statistics and correlations (Study 2).

(A) Choice condition: Autotelic					
Construct	M	SD	r		
Maximizing	4.14	1.17	1		
Reactance	2.88	1.29	0.36 **	1	
Algorithm adoption intention	4.62	1.57	0.09	−0.41 **	1
(B) Choice condition: Instrumental					
Construct	M	SD	r		
Maximizing	4.56	0.94	1		
Reactance	2.95	1.61	−0.10	1	
Algorithm adoption intention	4.60	1.72	0.04	−0.51 **	1

** *p* < 0.01.

**Figure A1:**
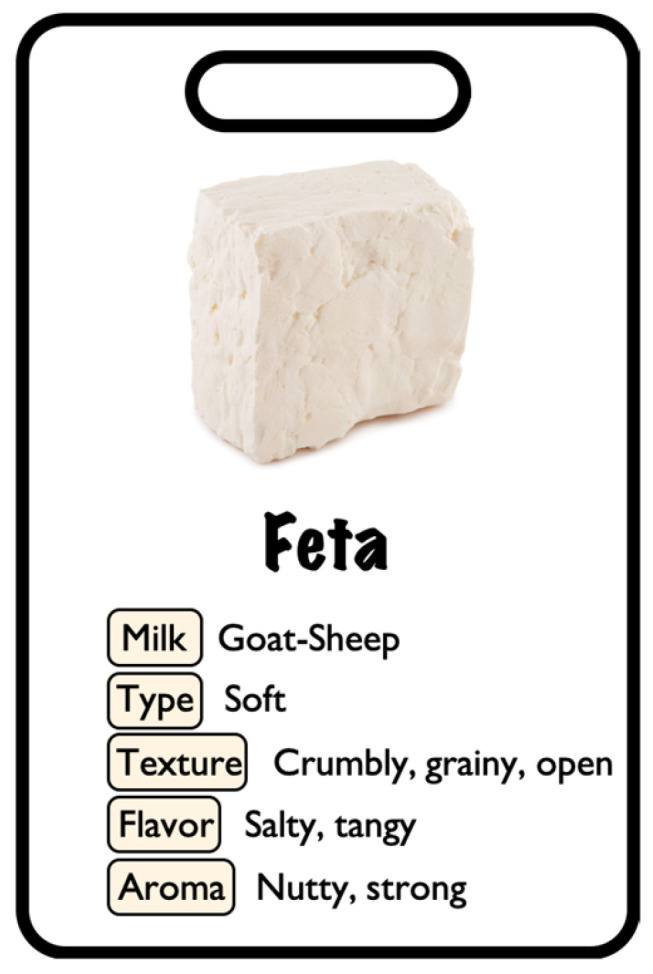
Example of a cheese option (Study 2).

## Data Availability

The data presented in this study can be obtained upon request by contacting the corresponding author.

## Appendix A

Maximizing Tendency Scale [28]

1. No matter what it takes, I always try to choose the best thing.
2. I don’t like having to settle for “good enough”.
3. I am a maximizer.
4. I will wait for the best option, no matter how long it takes.
5. I never settle.
6. No matter what I do, I have the highest standards for myself.
7. I never settle for second best.

Reactance scale (adapted from Kokkoris [25])

Because of algorithm-recommended options…

1. I often feel that I have limited choices as a consumer.
2. I often feel very restricted as a consumer.
3. I am often frustrated that I am unable to make free consumer choices.
4. I am often distressed that I could not have what I wanted as a consumer.
5. I am often irritated that many free consumer options are no longer available.
